# Airway Compromise due to Retropharyngeal Emphysema–A Rare Complication of an Extravasated Peripherally Inserted Central Venous Catheter

**DOI:** 10.1155/2019/6980475

**Published:** 2019-10-16

**Authors:** Ana Licina

**Affiliations:** Austin Health, 145 Studley Road, Heidelberg, Victoria 3084, Australia

## Abstract

A 48-year-old woman was scheduled for flexible bronchoscopy, video-assisted thoracoscopic surgery and mediastinal washout. She had developed voice changes, difficulty swallowing, shortness of breath with a fever and increased respiratory rate in intensive care unit 12 days after a double liver and kidney transplantation. Computerised tomography of neck and chest demonstrated extensive retropharyngeal and subcutaneous emphysema, laryngeal distortion and pneumo-mediastinum; however, the causative factors were not immediately obvious. Intraoperatively, an un-anticipated diagnosis of extravasated peripherally inserted central venous cannula (PICC) was made. Total parenteral nutrition had extravasated into the mediastinum and thorax. Subsequent inflammation and infection resulted in air pocket formation. The retropharyngeal air pockets were caused by mediastinal emphysema tracking through the tissue planes to the anterior and posterior larynx. Awareness of the tip position and accompanying clinical and radiological enquiry, must be performed prior to use of PICC lines in critically ill patients.

## 1. Background

We present the case of a 48-year-old woman who had received a liver and kidney transplant for severe autosomal dominant polycystic kidney (ADPKD) and liver disease (ALPLD). Mutations in one of two genes, *PKD1* or *PKD2*, account for most cases of ADPKD. Often, the ADPLD is associated with mild ADPKD. This patient had features of severe phenotype of both ADPLD and ADPKD. She required a dual organ transplant for the co-morbid dysfunction. Often the post-organ transplant patients require admission and lengthy supportive hospital care. During the course of her postoperative care she received a peripherally inserted central venous catheter (PICC) for the purpose of delivering total parenteral nutrition (TPN).

**Figure 1:**
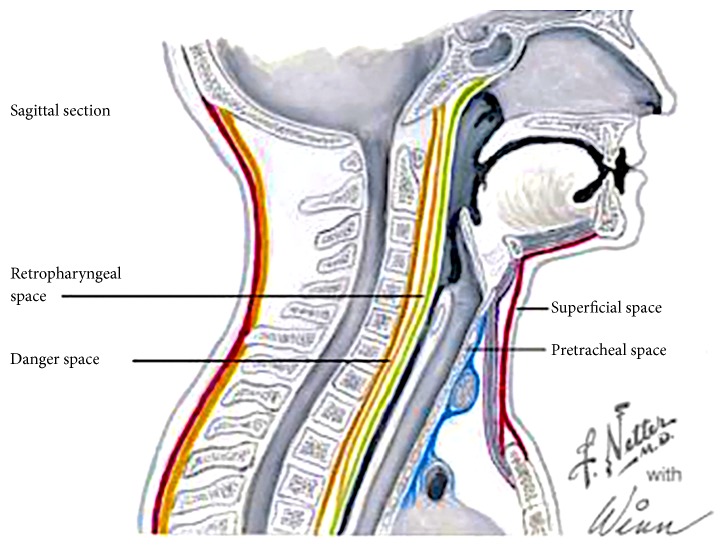
The spatial relationships of the true retropharyngeal space and the more posterior danger space. Danger space provides a channel of communication between the mediastinum and the retropharyngeal area. (Adapted from Intechopen under Creative Commons Attribution 3.0 License from https://www.intechopen.com/ open publishing)

Peripherally inserted central catheters (PICC) are a widely used form of central vascular access, both in hospital and community settings. The range of indications is broad, including the need for long-term antibiotics, total parenteral nutrition, chemotherapy treatment, blood products or blood sampling [1, 2]. They can be left in-situ for several months [3]. In addition to the high utility, these intra-vascular catheters have a favourable economic profile [3]. There is a perception of lower risk of PICC related complications, in comparison with central venous catheters, despite recent meta-analysis demonstrating otherwise [4]. PICC lines themselves are frequently used as “safer” alternative to centrally inserted venous catheters. Complications of PICC lines include malpositioning of the catheter tip, thromboplebitis and catheter dysfunction [5]. In particular, the malpositioning of the catheter tip can result in vessel rupture and extravasation. The extravasation of the infusate content can lead to mediastinal and intrathoracic complications. This patient had suffered an extravasation of the PICC line resulting in spreading of the hypertonic TPN into the mediastinal and thoracic cavities. Total parenteral nutrition is a hypertonic solution rich in lipids, protein and glucose. In cases of extravasation, it provides a substrate for inflammation and infection [6]. Mediastinal infection can result in formation of inflammatory contents and air pockets [7]. In this case, the contents in the mediastinum tracked through the retropharyngeal space to surround the airway structures. This tracking of contents from the mediastinal to retropharyngeal space occurs due to the anatomical connection between the two spaces. The retropharyngeal space is an anatomical region that spans from the base of the skull to the mediastinum [8]. The retropharyngeal space consists of the true retropharyngeal space and “the danger space”. The danger space courses more inferiorly than the true RPS, running into the posterior mediastinum until the level of the diaphragm (Figure 1) [8, 9]. Airway complications can occur due to the spread of pathological processes from the mediastinum through to the retropharyngeal space. If a differential diagnosis and clinical reasoning, are not broadened, rare but easily treated complications can be missed due to anchoring biases. Airway complications can have high morbidity and mortality, particularly if not considered or anticipated, in association with the performance of a perceived benign procedure such as insertion of a PICC line. This case highlights the importance of multidisciplinary teamwork between the surgical, intensive care and anaesthesia teams. It also illustrates the collaboration required to safely diagnose and manage difficult clinical problems occurring in patients undergoing complex medical care. To our knowledge, this is the first case description of an extravasated PICC line causing consequent airway compromise.

## 2. Case Presentation

**Figure 2:**
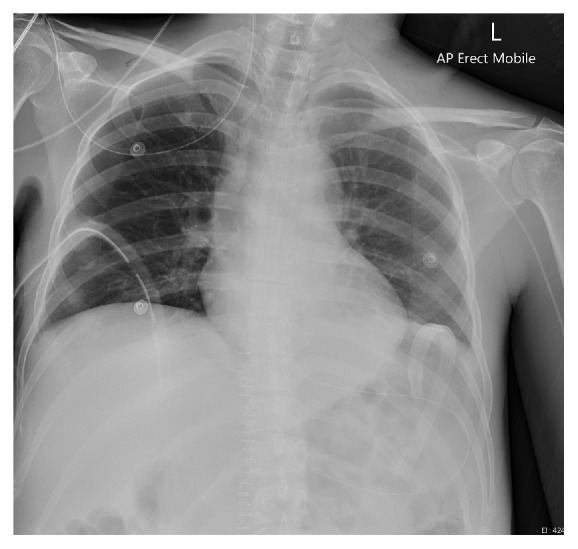
Chest X-ray showing the PICC line in the subclavian vein. Also shown is the R sided chest drain in situ.

A 48 year-old woman was scheduled for an urgent diagnostic flexible bronschoscopy, mediastinal washout as well as video-assisted thoracoscopic surgery. She was cared for in the intensive care unit having received a dual liver and lung transplant twelve days prior to the request for the thoracic procedure. The initial dual organ transplant (day 0) was complicated by intra-thoracic placement of the veno-venous bypass cannula. This was detected at the time and managed by resiting the cannula surgically into the axillary vein. The immediate post-transplantation course consisted of an initial uneventful recovery, monitoring and supportive management in intensive care unit. There was evidence of near complete resolution of the right-sided haemothorax with radiological confirmation on computed tomographic (CT) chest scan. Patient was afebrile with clinically stable markers of respiratory and haemodynamic function. She was extubated on day 9 post dual organ transplantation. The PICC line was inserted through the left basilica vein for total parenteral nutrition post extubation also on day 9. Both PICC lumens were found to flush and aspirate blood when tested clinically. Subclavian vein PICC tip placement was confirmed with a chest X-ray and total parenteral nutrition was commenced (Figure 2). Patient developed mild voice changes and shortness of breath 36 hours later. The PICC line was checked. No blood could be aspirated. The PICC line was deemed likely blocked and remained in situ. This decision was made due to the high confidence in the initial appropriate placement of the line. Two days following the PICC line insertion, there were further changes with alteration in the quality of voice and difficulty verbalising. Patient developed difficulties with swallowing. Clinically, there was attendant deterioration in the respiratory function with chest pain, tachypnoea and hypoxemia. Patient oxygen saturation was low in the range of 91–93% despite oxygen therapy via hudson mask. Increased respiratory rate was noted, with 18–25 breaths per minute. An urgent CT angiogram and barium swallow were performed in order to assist with the diagnostic process.

**Figure 3:**
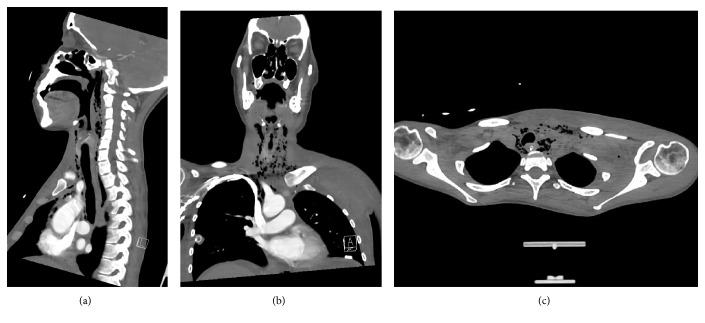
(a) Sagittal, (b) coronal, and (c) horizontal: computerised tomography slice demonstrating extensive retropharyngeal emphysema and pneumo-mediastinum.

**Figure 4:**
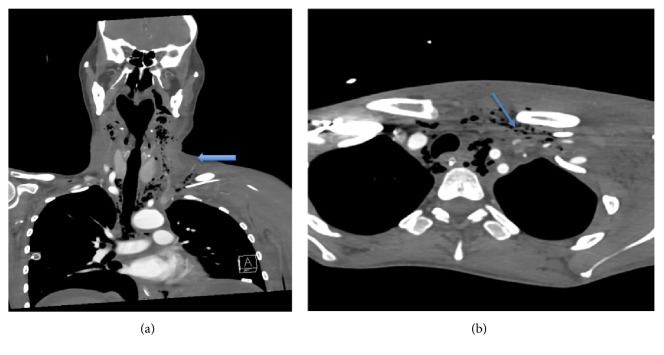
(a) coronal slice and (b) horizontal slice. Arrows demonstrating computerised tomographic angiography.

A Computerised Tomography scan of neck, chest and abdomen performed on day 12 following initial transplantation demonstrated mediastinal collection, mediastinal emphysema as well as extensive retro-pharyngeal air tracking (Figures 3(a)–3(c)). The CT chest and neck vessel angiography report noted that PICC line was not convincingly intravascular and appeared to be outside the great vessels (Figure 4). Left sided supraclavicular tissue streaking was noted. A barium swallow performed was thought to demonstrate a communication between the oesophagus and the trachea. It was thought that this may indicate the presence of a tracheo-oesophageal fistula as a potential causative factor of intra-thoracic complications. Arterial blood gases demonstrated significant hypoxemia despite oxygen therapy.

Patient returned to theatre for the planned procedure. Decision was made to maintain spontaneous ventilation during the flexible bronchoscopy, whilst the surgical team examined the trachea with a Storz 6.1 mm flexible bronchoscope. Sedation was achieved with 20 micrograms of Fentanyl and low dose of target controlled infusion of propofol. Oxygenation was maintained with 30 L/min at 100% High Flow Nasal Oxygen (HFNO) using OptiflowTM during spontaneous ventilation. Through examination using flexible bronchoscopy, no connecting fistula between the trachea and oesophagus was noted by the surgical team. This negated the most likely differential of tracheo-oesophageal fistula. It was thought that the small amount of aspirate due to impaired swallowing was misinterpreted as a connection between the oesophagus and trachea. Decision was made to secure the airway and proceeded with simultaneous generation of other differential diagnoses and patient management. Patient was induced with a modified rapid sequence induction, using 200 mg of fentanyl, 40 mg or propofol and 50 mg of rocuronium through a peripheral intravenous cannula. Oxygenation during the apnoeic period was maintained using the OptiflowTM device with THRIVE technique (transnasal humidified rapid-insufflation ventilatory exchange). High flow humidifies oxygen was delivered at 60 L/min at 100% oxygen and jaw thrust was maintained for ongoing airway patency [10]. CMACr videolaryngoscope standard blade 3 was used for direct and indirect laryngoscopy. Grade 1 laryngoscopic view was obtained on direct and indirect visualisation of the laryngeal structures. Oedema of the false cords was noted. Airway was secured with a Mallinckrodt 35 French left-sided double lumen endotracheal tube. Following a further review of the CT-angiographic images, mal-positioned PICC line was queried as a causative factor in mediastinal and thoracic pathology. Air pockets and tissue streaking were noted on CT-angiogram at the exit site of the PICC line from the subclavian vein.

Upon further documentation review, a temporal relationship was noted between PICC line insertion, commencement of TPN and the development of airway compromise with mediastinal symptoms. No blood could be aspirated through the catheter by the anaesthetist at the time of surgery. The PICC line was removed from the left basilica vein and sent to pathology for microbial screen and culture. Video-assisted thoracoscopic surgery was performed. Mediastinal washout and drain insertion were performed. At the completion of surgery, the left sided double lumen tube was exchanged uneventfully over an 14 Fr Cook® Airway Exchange Catheter through the tracheal lumen to a standard size 7 endotracheal tube. The patient returned to intensive care with appropriate sedation and remained intubated until the following day. Resolution of mediastinal radiological signs was demonstrated. Patient was extubated the following day with a resolution of airway signs and symptoms.

## 3. Discussion

This case illustrates a rare airway life-threatening complication of a PICC line, superimposed on a background of underlying complex medical problems. This is the first case report of an airway compromise secondary to air tracking from the mediastinum into the retropharyngeal space, where the causative factor was extravasation from a PICC line. The unique features include uncommon pathology superimposed on a background of a critically ill patient, posing complex decision making and teamwork challenges. We have described a rare pathology and a rare clinical consequence.

The most likely diagnosis of oesophago-tracheal fistula was excluded on diagnostic bronchoscopy. Following a clinical investigative pattern the correct diagnosis was then considered. This case illustrates the importance of generation of a wide number of differential diagnosis with a pattern of critical clinical reasoning. The positive predictive value of barium swallow in detecting oesophageal leaks is 59%; As such nearly half of the cases thought to have a leak, are false [11]. Through generation of multiple differential diagnosis, anchoring and fixation biases are avoided during the decision-making processes in order to achieve the best outcome for the patient. Given the complex clinical course of this patient, the differential diagnoses considered needed to be wide. At this stage, a malpositioned PICC line was not thought to be the cause of the mediastinitis, tissue emphysema, retro-pharyngeal air tracking and airway compromise.

The hypertonic total parenteral nutrition solution is rich in lipid, protein and glucose. As such, it can act as a causative agent of inflammation and infection in cases of extravasation [6]. There have been previous literature reports of total parenteral nutrition extravasation with consequent air bubble formation in the tissues [12, 13]. In a case report with a delayed migration of a PICC catheter into the mediastinum, extravasation of the TPN caused air pocket formation in the mediastinum in a similar fashion to the one described in our patient [12]. The authors in prior case reports did not hypothesize on the causative mechanism of tissue emphysema formation. The most likely cause of the tissue air generation is superimposed infection. Although the mediastinal fluid was not sent for pathological diagnosis, it is likely that sepsis in this patient was caused by mediastinitis. Superimposed anaerobic infection would account for air formation. Radiological features of mediastinitis include air-fluid levels, subcutaneous mediastinal or pericardial air [7]. A less likely causative factor, may have been inadvertent iatrogenic introduction of tissue air, through repeated attempts to “unblock” the PICC line when no blood could be aspirated. In clinical cases where the PICC line had extravasated into the pericardial sac itself, followed by the infusion of high volume intravenous fluid, pericardial tamponade with variable mortality has been reported [14].

Mediastinitis can be a challenging diagnosis to deduce, as it presents with a nonspecific confluence of signs and symptoms. It is often a diagnosis of exclusion with a wide and varied differential diagnosis. Signs and symptoms can consist of chest pain, dyspnea, hypoxemia, neck swelling, difficulty swallowing, dilated superficial and neck veins. Differential diagnosis of mediastinitis consists of number of alternative high morbidity conditions such as aortic dissection, pneumonia, pleural effusions, mediastinal masses or abscess, pericardial effusion or tamponade. The non-specificity of clinical signs and symptoms may lead to a delayed diagnosis or misdiagnosis, which contributes to patients' morbidity and mortality [8]. In this patient, mediastinits was caused through the leakage of the hypertonic TPN into the cavity. Following the mediastinal washout and institution of appropriate broad spectrum antibiotic therapy, the sepsis symptoms resolved.

In this patient positioning of the PICC line was confirmed with a plain chest radiograph on the day of the insertion to be in the left sided subclavian vein. This is not considered an ideal location by USA FDA guidelines [15]. This smaller vessel is more prone to the extravasation due to erosion by the hypertonic total parenteral nutrition solution. Following the initial aspiration of blood, hypertonic TPN solution was commenced. The following day, patient started developing voice changes and shortness of breath. The retropharyngeal space extends from the skull base to the upper mediastinum, lies posterior to the pharynx and esophagus, and is anterior to the prevertebral musculature [9]. The retropharyngeal space is divided into two anatomical areas by the very thin alar fascia- the anteriorly positioned true retropharyngeal space and the posteriorly situated danger space. The true RPS extends from the clivus inferiorly to a variable level between the T1 and T6 vertebrae. The danger space extends further inferiorly into the posterior mediastinum to the level of the diaphragm and is named as such because it provides a conduit for spread of infection from the pharynx to the mediastinum. It is through this space that pathological processes such as infection, cancerous cells or emphysematous pockets can spread. PICC's are being increasingly used in critically ill patients. The insertion site is perceived to be of lower risk as it minimises the risk of pneumothorax, haemothorax and can be used with greater degree of safety in coagulopathic patients. Some authors suggest that PICC lines may be more favourable in patients with severe cardiorespiratory abnormalities and morbid obesity [16]. Challenging the clinical perception that peripherally inserted central venous catheters may be safer as compared to central venous lines, a recent review found that malpositioning of the catheter tip, thrombophlebitis and catheter dysfunction were more common than with central venous lines [17].

There are strategic cogent airway guidelines available for managing a difficult airway [18]. For the diagnostic flexible bronchoscopy, a spontaneously ventilating technique was chosen. In case of a tracheoesophageal fistula, providing a conduit for tidal volume escape, spontaneous ventilation is considered a safer airway strategy. Trans-nasal humidified oxygen has been found to decrease the respiratory distress in Intensive Care Patients breathing spontaneously and undergoing bronchoscopy [19]. In the recent Difficult Airway Society Guidelines, the use of transnasal high-flow oxygen has been recommended in the setting of airway management. Nasal Oxygenation During Efforts of Securing A Tube (NODESAT) has been shown to extend the apnoea time in obese patients and patients with a difficult airway [10, 20, 21]. Apnoeic oxygenation techniques are now a key feature of the initial airway plan in a high-risk patient [22, 23]. We used the Transnasal Humidified Rapid-Insufflation Ventilatory Exchange (THRIVE) technique during rapid sequence induction. This is in accordance with the latest guidelines as published by the Difficult Airway Society. The role of videolaryngoscopy has achieved recognition as a key component of difficult airway management [18]. We utilised the rigid videolaryngoscopy technique as a blade of first choice. In our case the CMACr videolaryngoscope was the first blade of choice in order to perform laryngoscopy. We used the Mac 3 blade option in order to objectively grade the direct view, yet have the assistance of an indirect screen view. In addition, we chose to minimise the number of laryngoscopic attempts at securing the airway by utilising the indirect rigid video technique.

## 4. Conclusion

Mediastinal space is intimately connected to the airway tissues through the retropharyngeal space and danger space. Any mediastinal pathological processes can extend to the airway through the anatomically connected tissue planes. Migration, misplacement and potential catheter pericardial migration need to be considered in the differential diagnosis of intra-thoracic pathology including pneumo-mediastinum with retropharyngeal air tracking, subcutaneous emphysema, mediastinitis, pleural complications and pericardial tamponade. Clinical and radiological enquiry as to the PICC line integrity and position should be performed both as part of a differential diagnosis and prior to PICC line use in critically ill patients.

When unanticipated clinical deterioration occurs in a patient, iatrogenic causes must be considered. This is particularly important in complex patients requiring multiple interventions. Strategic airway management involves following not only the appropriately chosen guideline for the situation but working closely with our surgical colleagues as the differential diagnosis evolves through the intraoperative process.
